# Tourism and Sustainability in Times of COVID-19: The Case of Spain

**DOI:** 10.3390/ijerph18041859

**Published:** 2021-02-14

**Authors:** Libertad Moreno-Luna, Rafael Robina-Ramírez, Marcelo Sánchez-Oro Sánchez, José Castro-Serrano

**Affiliations:** 1 Department of Business and Sociology, Universidad of Extremadura, 10071 Cáceres, Spain; limorenol@alumnos.unex.es (L.M.-L.); msanoro@unex.es (M.S.-O.S.); 2 Department of Art and Land Sciences, Universidad of Extremadura, 10071 Cáceres, Spain; josecastro@unex.es

**Keywords:** COVID-19, tourism, sustainability, Spain, seasonality, domestic tourism, residents, economy, society, environment

## Abstract

The aim of this paper is to study the effects of the spread of the COVID-19 virus in different regions and its impact on the economy and regional tourist flows. To this end, the researchers have been guided by a set of propositions which they have tried to demonstrate with the results obtained. This research shows that the impact of the pandemic is still being evaluated. The analysis of the relationship between the tourism sector and the pandemic outbreak in Spain provides an instructive case study to assist tourism in its recovery process. The paper delves into the impacts on the main Spanish touristic regions during the pandemic and providing implications for tourism recovery. In Spain, the tourism sector is of major economic importance, becoming one of the most vulnerable countries when crisis affects this industry. The negative image of the country due to the high infection rates has had a negative impact on travel and tourism. The Balearic Islands have been the most affected region with an 87% decrease in tourist visitors. The trips made by Spanish residents inside the Spanish territory shows the first increase found in the series analyzed. Domestic tourism not only represents an opportunity for all regions in this critical situation, but the types of accommodation also play a key role.

## 1. Introduction

The world is experiencing an unprecedented global health, social, and economic emergency due to the COVID-19 pandemic [1,2,3]. On 11 March 2020, the World Health Organization (WHO) urged governments worldwide to prepare for the first wave of public health emergency. Nationwide lockdowns as one of the drastic measures were stablished in many countries to limit human interaction at close distances [4,5,6]. According to the WHO Coronavirus Disease Dashboard [7], the current outbreak has confirmed more than 61 million cases worldwide and caused more than 1.5 million deaths, remaining a devastating pandemic [8] with numbers that continue to grow [9].

Recent data from WHO [7] shows the United States as the country with more confirmed cases, followed by India and Brazil. Inside the European continent, the Russian Federation has been hit the hardest by the virus. France, Spain, United Kingdom, and Italy also shows a high number of confirmed cases. Nonetheless, there could be many more cases that have not been reported [1,10].

The COVID-19 outbreak has led to an unprecedented crisis in Spain. After China passed their peak in infections, the spread of the virus in Italy was quick, followed by Spain which became the second epicenter in Europe by number of cases, reaching one of the world’s highest mortality rates [11]. Since September 2020, a new wave of cases appeared, and many destinations have reintroduced lockdowns and travel restrictions [12,13]. Currently, according to data reported on 29th November [7], Spain is one of the leading countries of the so-called second wave with 1,628,208 cases and 44,668 deaths in a population of about 47 million people.

At the beginning of the pandemic, most of the research focused on the medical aspects. Now it is important to study the social, economic, and environmental aspects that this crisis has caused, and how it is affecting sustainable development [14]. It is necessary to analyze the consequences on society sustainability, both in the short- and long-term, so it can be addressed in the right direction.

Since the first sustainable development concept established by the Bruntland Report [15], three decades of discussion about sustainability have clarified that it does not exclusively concern environmental issues, but it is also necessary to create quality of life for the present and future generations, involving the social and economic field [16,17,18]. To stimulate collective action in balancing the three dimensions of sustainable development, the United Nations [19] established in 2015 a global agreement between governments ’Agenda 2030’, which comprise 17 Goals (SDGs) in areas of critical importance for humanity and the planet to accomplish in the next ten years.

Every aspect of our lives has been affected [20]: How we live and communicate with each other, how we work and travel [21]. The exceptional measures imposed by governments, rendered our streets empty, producing a negative impact on tourism and leisure [2,22].

According to data published by UNWTO in January [23], global tourism provided almost US$1.5 trillion of tourism expenditures and reached 1.5 billion tourist arrivals in 2019. Tourism has been affected on both travel supply and demand [20]. According to data on the World Tourism Barometer [3] published in July 2020, the lockdown imposed led to a 98% decline in global international tourist arrivals this year during April and May, as compared to same period 2019, and 70% between January and August. The cost up to May was more than three times the loss during the global economic crisis of 2009, and this is translated into a fall of 300 million tourists leading to a US$320 decrease in tourist spending. For all these reasons, the UNWTO [24] qualifies 2020 as “the worst year in the history of tourism”. This source estimates the drop in international arrivals at 74%, with a loss of more than one billion fewer arrivals globally, while a recovery in activity is not expected until at least 2022.

Tourism plays a crucial role in many countries’ development. Many of the countries most affected by the health emergency, like Spain, France, or Italy, are key destinations globally [25]. The impact of the crisis will be particularly critical on these territories due to its heavy dependency on international tourism, making them more vulnerable [20,24,26].

According to the World Tourism Organization, Spain is the country with the second highest international tourist arrivals figures [3]. This dependence on tourism, which represents 12% of the economy’s GDP, makes it the third most vulnerable destination in the world, in comparison to the rest of the tourism leading countries [27]. Furthermore, together with the heavy fall in occupancy rates this year, shows the importance of carefully studying the Spanish case. The purpose of this investigation aims not only to understand the gravity of the situation experienced by the country, but to provide measures on the recovery of the tourism sector.

Spain was chosen for this case study as is one of the most visited countries in the world, with more than 83.7 million tourists per year. Is the second most visited country after France, and the second in tourist expenditures after the United States, with more than 112,319 million US$ [28]. In Spain, the tourism sector is of major economic importance, as it generates more than 2.6 million jobs, comprising 12.8% of the country’s total employment, and contributes 12.3% to its GDP [29].

The deep and quick changes are having a devastating impact on the economy and employment globally [2]. Current estimations establish that more than 100 million jobs are at immediate risk only in the tourism sector [30], one of the most labor-intensive sectors of the economy, and more than half of the workers are women [21,31]. This threatens to roll back progress made in advancing the Sustainable Development Goals (SDGs) of Agenda 2030, and losses from billions to trillions in export revenues from tourism [3,32].

This crisis is affecting the tourism industry in an unprecedented scale. Currently, there is a clear lack of research on how crises affect this sector. To study this gap, a spatial and temporal analysis of the impact of COVID-19 on tourism across the Spanish regions has been carried out in the present paper. The main goal is to study if the spread of the virus throughout different regions has impacted differently to their economy and tourist flows. This question is particularly relevant given the importance that tourism has for the country economy and especially for some regions.

To achieve this objective, six research questions or proposals have been formulated, referring to the effects of the crisis in the Spanish regions. These research proposals have been formulated according to the exposure of each region to the different tourist flows, as well as according to the degree of affectation by the pandemic and the effect on employment in the tourism sector.

The paper is structured as follows: Section 2 provides an overview of the impacts of the pandemic on the environment, the consequences of this crisis at a societal level and the repercussions of the measures imposed on the economy. Section 3 follows with an analysis of the impacts on the tourism industry. Section 4 describes the objectives, materials, and methods used in this research, followed by a study of the current Spanish situation on all these pillars in Section 5. Section 6 and Section 7 provide the discussions and conclusions, suggesting some implications for the recovery of the Tourism sector in Spain.

## 2. COVID-19 and the Environmental, Social, and Economic Impacts

This article refers to the impacts that the pandemic crisis is causing in different sub-systems; in particular the environment, the social and economic systems, within the latter we especially include the tourism sector. Given that the objective of our work is to analyze how the spread of the virus has had several consequences depending on its territorial distribution (autonomous regions) in Spain, it is necessary to start from a general evaluation of these impacts.

Environment change is one of the biggest and most vital challenges for humankind. Air pollution deeply affects people’s health, increasing the severity of chronic diseases and aggravating the symptoms in coronavirus cases [2,33].

The confirmation of the confinement’s positive effect on the environment and of mobility restrictions has become more evident in certain areas of the planet where there was an accelerated industrialization. This might be the only positive side of the pandemic. Self-quarantine and social distancing for more than two months, and subsequent confinement measures in the second and third waves, in the words of Lokhandwala and Gautam [34], has given nature a “healing time”, due to the reduction of the human impact on the natural environment. These authors confirm a great impact of the containment measures on air quality, which is being experienced by all and recorded in several official reports, about India. It has given way to blue skies in cities like Delhi, marine life is experiencing higher activity, pollution levels have decreased in almost all metropolitan cities, and animals, particularly birds, seem to enjoy greater freedom. It further notes that, in metropolitan cities like Delhi, where the energy footprint was high, currently has improved air quality on a larger scale [34].

As human activities were restricted in most of the countries, a reduction of more than 50% of atmospheric pollutants were seen in some cities, improving air quality and helping to improve public health in countries such as France, Germany, Italy, and Spain [35,36]. This showed clean air and transparent skies, having little impact helping to recover the ozone layer though, for a significant change, there should be a mind shift in all countries’ economies [37].

Additionally, ecosystems are recovering greatly, and many beaches around the world are being cleaned up due to a reduction in the waste usually generated by tourists [38]. On the other hand, among the negative indirect effects is the increase in domestic and medical waste. The online food ordering has is on the rise, consequently, waste generated by households has increased. Furthermore, restrictions to recycle waste in countries like the USA and Italy has been another negative indirect effect of the health crisis [37].

From the social impact’s perspective, this pandemic has been unprecedented because of its evolution from a health shock to a humanitarian and development crisis [32,39]. The virus has already brought significant challenges all over the world and people continue to suffer from both the health and the economic shock [9,11], as impacts on the economy are pushing workers and their families into poverty without income or social support [21].

Consequences of the pandemic outbreak have caused a negative gross domestic product (GDP) and increased inequality and poverty [40]. Millions of people have lost their jobs at extremely high rates [41]. The sectors with higher unemployment increases are those like hospitality sector, as demand for these services has ceased to exist [42]. Least qualified workers are more vulnerable, as their work situation does not allow the possibility to work from home [43], having a higher probability of unemployment and exposure to infection [41].

Hospitality has a majority female workforce, representing globally between 60 and 70% of the workers according to ILO [44]. Therefore, the pandemic is increasing existing inequalities, disproportionately hitting women and the most vulnerable population groups [30,45]. This also affects workers with precarious jobs, pushing an additional 71–100 million people into extreme poverty, reaching 684 million globally [13,21,46]. 

In most vulnerable households, income often depends on one person, increasing the risk of the whole household falling into poverty and social exclusion [43]. The situation for single parents, 78.4% of them are women, it is especially difficult if they struggle with work and caring for children when schools are closed [21].

The Internet has become the main method to obtain essential supplies and receive essential services [20,42] but not everyone has access to technology or the capabilities to use it; therefore, this situation will influence how communities fight the crisis [22].

The education system has been affected in an unprecedented scale. At least 147 countries, have moved their school and universities’ courses online [39], affecting more than 1.4 billion students of all levels of the education system [30]. Lack of access to the technology needed for learning at home [47] leads to limited access to online learning, where only families with access to technology will ensure that education continues during social isolation [20]. This situation warrants human development to decline worldwide for the first time in 30 years, and the first increase in global poverty since 1998, reversing all of the progress made [41,45].

The pandemic is putting gender equality in jeopardy, threatening fragile gains on human’s rights [32]. Globally, women, on average, perform three times as much unpaid care and domestic work as men, a situation that intensifies with school closures and when health systems are overloaded [30]. Furthermore, lockdowns affect vulnerable people, since the outbreak, violence and sexual assault against women and girls is on the rise, resulting in a shadow pandemic [21,39].

COVID-19 is not just affecting people’s physical health, high rates of mental distress, including stress and depression are reported, with women reporting higher rates than men [42]. Increases in social disparities, job uncertainty, income loss, and an increase in gender violence are some of the reasons for the need of mental health care [48].

Tourism sector is an important driving force for inclusive socio-economic development as contributes to job creation both directly and indirectly, particularly for women and young people [49]. Actions should not jeopardize the fragile gains that have been made on gender equality. Progress on Agenda 2030 depends on a common response that builds equal and resilient societies for the future [32].

Regarding the economic impacts, the COVID-19 pandemic and its mobility restrictions has brought severe socioeconomic consequences [4,20]. It has decreased consumption and demand, affecting communities, businesses, and organizations globally [9,10,50,51]. The great uncertainty of the pandemic has caused markets’ disruption and high economic costs on an unprecedented scale, making them highly unpredictable [11,36,52,53].

For the first time, countries worldwide are experiencing negative figures in their GDP due to lockdowns measures [38,41]. According to the last World Economic Outlook [54], global economy is showing unprecedented figures in recent history, shrinking by −4.4% this year. Asia will have the first regional recession in almost 60 years. The economies of the United States and Europe are projected to contract between −18% and −13%, respectively. The drop in Latin America and Arab States is estimated by −11.4% and −10.6%.

In the second quarter of 2020 GDP has decreased −13.9% in the European Union, in comparison with the same quarter of the previous year [55], showing the sharpest decline observed [21,22,23]. The third quarter 2020 already showed a recovery in comparison with the previous quarter, with positive growth for all the countries and only few exceptions. This might be due to the borders reopening and resumption of the economic activity in some sectors.

Spain registered the greatest GDP fall in Europe in the second quarter by −21.5%, followed by France (−18.9%), Italy (−17.9%), and Portugal (−16.4%), with the most representative figures [55]. The reason that these countries have experienced such an unprecedented decline might be due to the relevance that tourism sector has on their economies, as these countries receive the highest tourist flows in Europe. Therefore, the border closures and lockdown measures has been devastating for these countries.

Worldwide, the pandemic has led to a catastrophic hit to the global labor market. As supply chains disintegrate, whole sectors collapse, and most companies must implement an indefinite hiring freeze while other businesses have been forced to close [11]. The emergency measures adopted to contain the virus are having an uneven impact on workers from different occupations and industries [40]. According to the last ILO Monitor [56], workers around the world are facing unemployment and loss of their incomes with 94% of them currently suffering some kind of closure measure in their countries or a decrease in working hours.

The global job losses are estimated to be 495 million in the second quarter of 2020, a considerably larger decline than the 195 million estimated in April for the same quarter [56], reflecting the worsening situation globally [21,22,23,24,25]. The International Labor Organization estimates a loss of 345 million jobs for the third quarter, a quite small improvement for this tremendous crisis.

In addition, crises also accelerate technology innovation, while some businesses are struggling, other are thriving [42]. Due to fear of infection individuals have changed their consumption habits, resulting in an online overconsumption [20,22,40]. Working via digital means only selected parts of the economy will remain open [41]. The survival of many companies depends on their ability to adopt different forms of e-commerce [39]. The consequences of the pandemic might help towards teleworking and a greater use of the Internet [50,51].

It is necessary to mention that although some destinations and tourist attractions have created virtual visits, the tourism sector cannot develop its full social, cultural, and economic potential, solely on the basis of online offers, like other sectors of the economy can. By definition, tourists should be able to travel, because the essence of tourism is personal and direct experiences in destinations.

Tourism businesses have been racing to ensure the safety and re-design experiences to re-start tourism [22]. Innovative actions are key for tourism business as they often suffer from innovation deficiencies [31].

Tourism is firmly positioned in the 2030 Agenda for Sustainable Development, it has been identified by the United Nations Environment Programme (UNEP), as one of the sectors that can lead the transition to the green economy [44]. Its importance as a driver for job creation and the promotion of local economic development is reflected in the Sustainable Development Goals 8, 12, and 14, which include tourism-specific targets [49,53].

Governments and destinations have been providing stimulus packages and interventions to ensure the continuity of tourism firms and jobs [22]. The European Commission has provided an unprecedented degree of economic support for the tourism sector. However, this situation does not seem to likely to recover any time soon, with some regions of the world remaining closed and the pandemic continuing to spread [57].

## 3. Tourism in the COVID-19 Crisis

The coronavirus pandemic has had the most significant impact on the tourism industry like no other previous event in history [22,38]. The restrictions imposed have brought international travel to a standstill, showing the vulnerability of the industry to crises [26,58].

Many airlines are already in bankruptcy due to the restrictions in domestic and international travel [41], decreasing by more than 90% during April and May 2020, compared to same period in 2019 [59]. Europe has been the most affected region, as the number of international flights dropped by 95%, from 576,572 in May 2019, to only 26,796 flights in May 2020 (−535,867). Followed by Asia (−207,556) and North Africa (−96,065). This has had a decisive impact on the accessibility of tourist destinations. June brought a slight recovery due to the lifting of strict border closures, but the difference with the same period last year was still exceptionally large.

**Figure 1:**
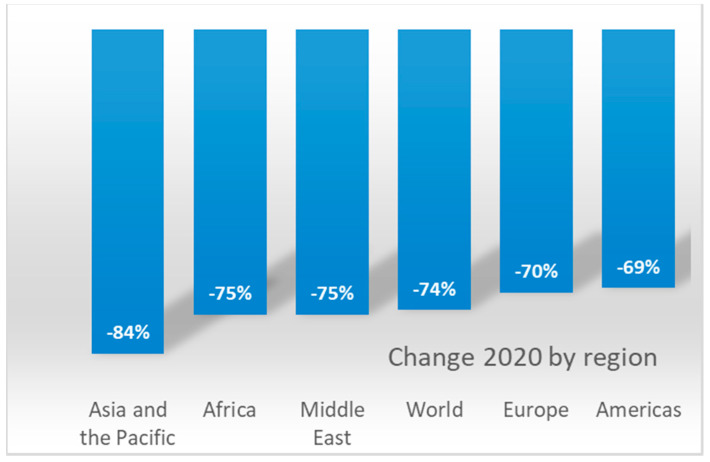
Tourists arrivals decrease in 2020 by world regions. Source: World Tourism Organization (UNWTO) January 2021.

The pandemic outbreak has caused the international tourism economy to contract 70% in the first eight months of the year, according to the last UNWTO Tourism Barometer published in October [57]. This translates into 700 million fewer international tourist arrivals in comparison to 2019 and a loss of US$ 730 billion from international tourism, more than 8 times the figures after the global economic crisis in 2009. International arrivals decreased 81% in July and 79% in August, traditionally the two busiest months of the year and the peak of the summer season. As Figure 1 shows, the UNWTO [24] estimates the drop in international arrivals in 2020 at a global level at 74%. The areas with the greatest reduction in international tourist arrivals were Asia-Pacific (−84%) followed by Africa and the Middle East (−75%), while Europe (−70%) and the Americas (−69%) would remain below the global average loss of international arrivals.

The major tourist destinations worldwide have experienced a strong impact due to the intense decline in international tourist arrivals. Spain was the second destination in international tourist arrivals in 2019, and this year has shown one of the largest decreases with a 98% drop in international tourist arrivals. Other European destinations, such as Great Britain, would have experienced smaller decreases, with a 54% less in June 2020. As for what was the third leading country in tourist arrivals in 2019, the United States has also experienced an intense decrease of 95% in June 2020 [57].

According to the latest data published by UNWTO [60] on hotel occupancy rates in September 2020 part of the leading destinations has fallen significantly. Spain is in fourth place of the top 10 list of tourist destinations with the highest occupancy rates, with 61% in January 2020. However, in September 2020 Spain registered an occupancy rate of only 27%, while other countries such as China (62%), United States (48%), Great Britain (46%), Turkey (45%), Germany (44%), France (42%), and Italy (34%) had much higher rates. Therefore, Spain has fall to the last position of the leading destinations in September 2020, both in international arrivals and in hotel occupancy rates.

The tourism sector has experienced a continued expansion and diversification during the last decades and is one of the most dynamic and fastest-growing economic sectors [44]. Travel and tourism accounts for 10.3% of global gross domestic product (GDP), a total of US$8.9 trillion [25]. After the global economic crisis of 2008, there was an employment growth in accommodation and food services up to 35%, exceeding the overall employment growth (11%) [61]. In 2019 the sector accounted more than 330 million jobs worldwide, equivalent to one in 10 jobs globally [49].

The significant multiplier effect on employment in related sectors cause a domino effect when the sector falls, including all those elements that depend directly or indirectly [26]. It is estimated that one job in the tourism sector creates about one-and-a-half additional or indirect jobs, like transportation, food and beverage provision, handicrafts, and the preservation of cultural and natural assets [44]. With the sudden halt of the economic activity, workers in the tourism industry are now facing devastating difficulties [20].

It seems unlikely that the sector will return to normalcy any time soon. Not surprisingly, fear of infection and risks of new lockdowns has a tremendous effect on tourists’ perceptions, increasing insecurity and making people reduce exposure voluntarily [22,54,60,62]. The pandemic might alter the image of destinations in front of potential tourists, in those territories that suffer from high infection rates [31]. Tourists will evaluate their travel decisions according to the measures being taken by destinations on this exceptional situation.

In view of supporting a safe restart of tourism, destinations are implementing safety and hygiene protocols measures [28]. During the first week of July, some countries started to reopen borders and a small resurgence in air traffic was observed mostly by the domestic market [49,59].

The impact of the current pandemic will likely last longer for tourism than for other affected industries. Despite the effects of crises on tourism, history verifies its own resilience [22,58].

## 4. Objectives, Materials, and Methods

The objective of this research is to analyze the effects of the spread of the virus in the different regions of Spain, as well as to explain its impact in a differentiated manner on the economy and on regional tourist flows. To this end, we start from the following premises or research hypotheses:

**Proposition** **1.** *The regions with higher number of cases will be the most affected by the pandemic.*

**Proposition** **2.** *The most touristic regions will be the most affected by the crisis.*

**Proposition** **3.** *The most infected regions will be the less visited by tourists.*

**Proposition** **4.** *The most touristic regions will be the regions with higher unemployment rates.*

**Proposition** **5.** *The regions with more COVID-19 cases will be the ones with higher unemployment rates.*

**Proposition** **6.** *Domestic demand is expected grow faster than foreign movements.*

Regarding the working materials for the development of this analysis, the first task is to illustrate the relationship between the tourism sector and the outbreak of COVID-19 in Spain. For this purpose, the regions with more confirmed cases were selected to study if there was any relationship between the impact of confirmed cases and the most tourist regions. The study aims to evaluate the impact on the supply side and demand of tourist services, which implies estimations not only on the impact that mobility restrictions may have caused on tourists’ movements, but also the contraction in the tourism services supply. From data obtained from the 17 Spanish regions located in the Iberian Peninsula.

Daily data from 31 January until November 2020 has been collected, selected, and organized from various official sources provided by national and international level institutions: mainly, the Survey of Tourist Movements at Borders (FRONTUR) [63], the Spanish National Institute of Statistics (INE) [29] and the National Epidemiology Center (CNE) [64]. Available data was raw and unclassified, therefore, in order to make the data available for the study required sorting, purge and classifying them. 31 January 2020 has been chosen as the starting point for this evolution study since it was the first case identified in Spain.

The overall impact of the pandemic on the environment, society, and the economy has been considered from various sources, all of them secondary. In the case of the impacts on the environment, on which there is still a scarce literature, five papers published in high impact scientific journals have been taken into consideration. The paper deals with the measures taken by EU countries against the pandemic. Energy deficits, highlighted by COVID-19, are explained by Bresemer et al. [33]. The analysis of the effects on air quality is addressed by Zambrano-Monserrate, Ruano, and Sánchez-Alcalde [37], and, for the European context, by Islam and Chowdhury [35].

Regarding the social impact of COVID-19, the sources used are based on a large volume of statistical information, obtained directly from national and international organizations or from other secondary studies. Up to 18 different references have been consulted on these issues, such as the effects of COVID-19 on human development levels, addressed by the United Nations Development Programme (2020) [39]. Regarding the labor market and working conditions, the information provided by the International Labor Organization [40] stands out, in addition to other references that we will collect throughout this work. To analyze the impact of the pandemic on the tourism sector, we have resorted to the ILO [30] and the International Monetary Fund [45]. Regarding the effects on education, the UNESCO report [47] has been analyzed, referring to the reasons why learning should be strengthened, and education funding protected, in the current pandemic situation. The interpretation of the situation and its social impact has been considered on the basis of works published in reference journals, some of which are: Palomino, Rodriguez, and Sebastian, [40]; Kanda and Kivimaa, [41]; Donthu and Gustafsson [42]; Lakner et al. [46]; and Cenat et al. [48].

Regarding the economic impacts, several documents have been analyzed. Among them we have taken as reference the following official data sources: World Bank [51]; CCSA (Committee for the Coordination of Statistical Activities) [21]; ILO (International Labour Organization) ILO Monitor: COVID-19 [56], and EUROSTAT [55] and UNWTO (World Tourism Organization): World Tourism Barometer—October (2020) [57].

As analysts of the current situation regarding economic impacts, authors such as Tobías [10] have been focused on perimeter closures and their economic effects in Italy and Spain. Regarding economic risks for the tourist sector several works are addressed such as Zhang, Hu and Ji [11]; Tisdell, [50], and Shehzad, Xiaoxing, and Kazouz, [53].

COVID-19 data corresponding to the provinces of Spain was downloaded from official sources of epidemiological information (World Health Organization, National Center for Epidemiology (CNE) [57] which allowed to assess the trends across regions. Health information for each region like number of daily accumulated cases and confirmed deaths were updated weekly. Nevertheless, due to the exceptional situation caused by the pandemic, the Spanish health system was not prepared to deal with this crisis and some regions were not able to give accurate daily data.

On the other hand, socio-economic and tourist data have been collected from the State Public Employment Service (SEPE) [65], National Statistics Institute (INE) [29], EUROSTAT [55], International Labor Organization (ILO) [56] and the United Nations (UN) [39]. Several variables have been considered for the analysis of the impact of this crisis in Spain: GDP of each region, unemployment rates, tourist arrivals variation rates (international and domestic tourism), and the supply of tourist services.

The data have been divided into three stages, with the beginning of the pandemic in January–May (period with more restrictions in Spain), the second stage from June–August (lifting of the state alarm), and September–November (the appearance new wave of cases and state of alarm).

Regarding the methodology, we have applied a comparative analysis, given that the objective is to carry out an analysis of the different Spanish regions or Comunidades Autónomas, which constitute the existing administrative division in Spain. This has been the objective pursued in the organization of the data and their presentation through tables, illustrative graphs, and maps.

Cartographic representation is of particular interest when the impacts have territorial and management consequences, as is the case of Spain in the present study. The procedures for comparison operations are quite varied. In the case of data representation by thematic maps, we have used geographic information systems for their representation in thematic cartography, with automatic classifications by means of natural breaks in the data.

We, therefore, consider that the use of the comparative method is an effective tool when it comes to describing complex events, which are important from a social, economic, or political point of view, and which have differentiated impacts in the different areas or territories.

## 5. The Case of Spain

**Figure 2:**
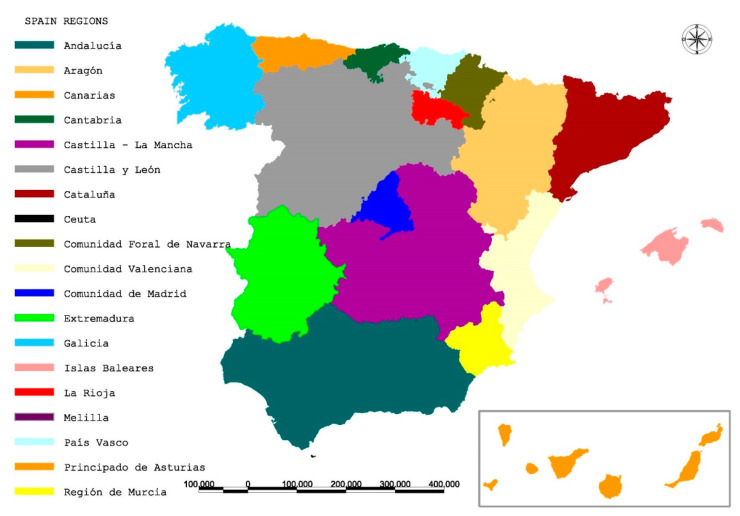
Spanish regions (Comunidades Autónomas). Source: Own elaboration.

The first confirmed case of COVID-19 in Spain was reported on 31 January 2020. It was an imported case corresponding to a tourist visiting the Canary Islands. After a month, the confirmed cases increased to 100 [66]. Despite the centralized measures to control the spread in the country, different impact across Spanish provinces have been observed as it is shown in Figure 2 [43].

### 5.1. Spanish Economy and the Labour Market

The Spanish economy has suffered a severe hit due to the pandemic. According to the National Institute of Statistics [29] the GDP in the last quarter of 2019 was 2%, while on the first quarter of 2020 Spain already showed negative figures, −5.24%, due to the state of alarm imposed on 12 March and the strict confinement measures. The consequences left the economy activity on halt, with limitations to only essential services for more than three months at the national level [43]. This resulted in a temporary layoff, decline in private consumption, companies closed, and with hospitals at their limits [67], which brought a contraction of −18.5% on the second quarter and is currently in recession.

Due to the reduction of mobility measures that were taken in Spain during the state of alarm, April and May were the worst months of the year for tourism, as airports and borders were completely closed to international tourists [68] until June 30 during the first wave. The entire hospitality industry was also closed during these months, with exceptions for those hotels that were offered as medicalized accommodations. It was only during this period that the borders remained totally closed to foreign travelers in Spain. Subsequently, there has not been a total closure again, but both tourist activity and the possibility of travel to Spain for foreigners from outside the European Union have been considerably restricted, especially since the state of alarm was re-declared on 25 October 2020. These restrictions are still in force today.

The fact that during the first wave the closure of tourist activity and borders was total, while it has been only partial during the second wave, constitutes one of the main differences for the evolution of the sector in both periods. In addition to this, we must clarify that during the first wave the restrictions were the same for all regions of the country, since the anti-COVID measures were adopted in a centralized manner by the Spanish government. However, during the second wave, the central government has taken a step back in decision-making, ceding more autonomy and responsibility to the Autonomous Regions, so that they can make different decisions regarding the restrictions to be imposed on the different sectors of economic activity, especially in the case of hospitality activities and the tourism sector in general. This will imply differences in economic and tourism activity during the third and fourth quarters of 2020 in Spain, but the absence of data for this entire period prevents us from addressing such analysis here.

The restrictions may affect economies in a different way just because their productive structure is not the same. When a certain economic activity is closed in a sector like hospitality, working is not possible. Countries who are specialized in these kinds of activities, like Spain, will suffer more from capacity and mobility limitations with enormous economic and social repercussions [40,43].

The impact on the Spanish labor market is unprecedented. Unemployment data registered by the Public Employment Statal Service (SEPE) [68] shows an 9.3% increase in March in comparison to the previous month, establishing the number of unemployed people at 3,548,312. These figures have never seen such an elevated increase, in one month all the progress made since the end of the previous economic crisis has been lost [43].

The unemployment rate in Spain have reached the peak in the second quarter with a 17.33% (3,862,883), the highest in Europe and one of the 10 countries with the highest unemployment rate worldwide [54,65]. Despite this negative evolution is still much lower than the one seen in the first quarter of 2013, with 26.94% during the earlier economic crisis. However, this numbers do not include workers under the Temporary Employment Regulation, known as ERTE, which is a measure implemented by the Spanish government to protect jobs and to reduce impacts in businesses and individuals. When business cannot afford to pay employees due to the cease of activity, employees under the ERTE measure will still receive a percentage of their salary [66].

SEPE [65] data shows an unequal impact among the population, with the majority of women (57.7%) unemployed in the hospitality sector. Furthermore, immigrants, temporary workers, and less-qualified workers would be some of the most affected groups with an increased risk of falling into poverty and social exclusion [43].

In 2019, Spain’s population was 47,329,981 million people, distributed among 17 regions and two autonomous cities (Ceuta and Melilla), which are further divided into provinces. Table 1 presents the current regions in Spain with the highest total unemployment figures, the unemployment in the service sector, the economic growth of each of these regions and the accumulated COVID-19 cases until the end of October 2020.

According to the National Epidemiology Center (CNE) [64] from January to July have been 321,561 cases registered in Spain. The regions with the highest COVID infections rate per 100,000 inhabitants are Andalusia, Catalonia, Valencian Community, Community of Madrid, Canary Islands, and the Balearic Islands. According to data from the Spanish National Statistics Institution, these regions are also the ones that usually have more tourist flows in Spain [63], therefore, they mostly depend on tourism and the service sector for their economy.

**Figure 3:**
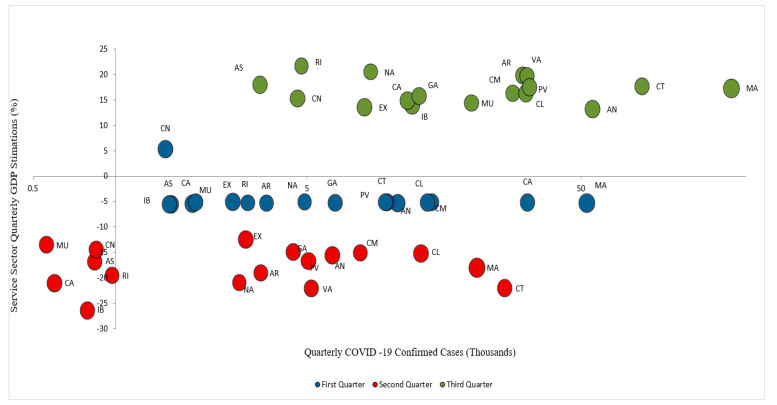
COVID-19 confirmed cases and GDP variations in Spain per region. AN: Andalucía; AR: Aragón; AS: Asturias; CA: Canarias; CN: Cantabria; CM: Castilla-La Mancha; CL: Castilla y León; CT: Cataluña; MA: Comunidad de Madrid; VA: Comunidad Valenciana; EX: Extremadura; GA: Galicia; IB: Islas Baleares; RI: La Rioja; NA: Navarra; PV: País Vasco; MU: Región de Murcia. Source: Own elaboration from SEPE, INE and CNE data.

The state of alarm established in Spain on 14 March 2020 has impacted these territories the most, showing a decrease between −21% and −26% of their GDP (see Figure 3). The other regions in Spain would show a recession around −15% [29]. They also show the highest unemployment figures, which encompasses 69.25% of the total Spanish unemployment in the second quarter. The service sector in Spain represents 70.80% of the total unemployment figures, in addition only these six regions, accounts for 70.03% of the total service sector unemployment at the national level.

In the first quarter, all regions showed a similar recession, but in the second quarter, the regions where the service sector predominates the most, showed a heavier decrease in their GDP: Canary Islands −21%, the Valencian Community −22.1%, Catalonia −22%, and the Balearic Islands −26.4% [65].

Although the Balearic Islands is the most affected region by the current crisis, showing the highest negative GDP figures (−26.4%), in comparison to the previous quarter of the year, the registered unemployment data does not show the same drastic figures. This may be due to the fact that the majority of workers from the Balearic Islands starts with the summer season, in April, and travel from the peninsula to work there, so when they register as unemployed, it would be in their community of origin, therefore, will not appear on the island records.

In July, a small improvement (2.32%) on the unemployment figures was seen due to the end of the state of alarm on 21 June and the reactivation of some parts of the economy. But currently, in the month of November, unemployed people are reaching the peak seen in June. This might occur due to the new wave of cases from September on, which surpassed the daily confirmed cases since beginning of the pandemic, reaching 22,610 confirmed cases in one day, on 27 October [64], which forced the government to establish a new state of alarm. This new measure is less severe than the first one, with curfews implemented nationwide and some capacity and mobility limitations, still, the unemployment figures from 2020 are 17% higher than the same period in the previous year.

### 5.2. The Economic Impact in Spanish Regions

An economic and health analysis is particularly important to understand the impact of changes in tourism at a national level, but it also necessary to study the regional components in order to understand the Spanish case. From January until July 2020, Spain received 13,249,637 international tourists, −72.4% fewer visitors compared to the same period 2019. Only in July, Spain received 75% fewer tourists in comparison to the same period last year [69].

From the six most touristic regions, the Canary Islands shows a decrease of 61.4% in the first seven months of 2020 in comparison to the same period of 2019. Catalonia is the second region with almost 2.8 million tourists and had a decrease of 75.6%. Andalusia, with more than 1.9 million in third place, shows a 72.3% decrease in 2020. The number of international tourists fell by 69.7% in the Valencian Community, and 67.1% in the Community of Madrid. The Balearic Islands have been the community most affected by the crisis in terms of decrease in tourist visitors −87% [69].

The first quarter shows that the 89.7% of the trips made by the Spanish residents were inside the Spanish territory, with a decrease of 30.7% compared to same period last year. In the second half of March, 370,000 trips were made, in comparison to the 8.5 million of the same period in 2019. The main destinations chosen are Andalusia (17.2%), Catalonia (12.9%) and the Community of Madrid (9.4%). On the other hand, trips abroad, which represent 10.3% of the total, decreased by 25.2% [70].

According to the Statistics of Tourist Movements at the Border [29,63], the total expenditure in the first quarter represents a decrease of 22.6% compared to the same period of 2019. Travels within the national territory shows a fall in total spending by 24.3%, and in those made abroad by 19.1%.

**Figure 4:**
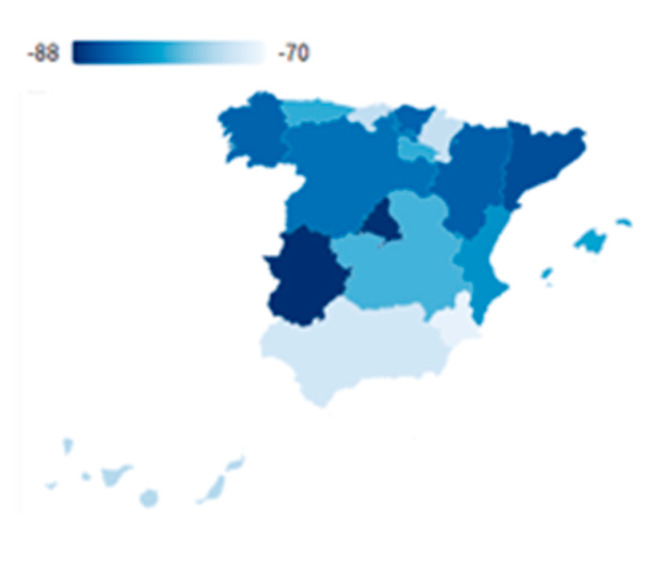
The inter-annual variation rates of tourist arrivals in the second quarter of 2020. Source: Own elaboration from INE data.

Figure 4 shows a high decrease in the annual variation rate of tourist arrivals in the second quarter of 2020 in comparison with 2019. However, every region shows different changes, therefore, is important to understand the economic and health context of each one. There is a high impact on tourist arrivals both in the Community of Madrid (−87.67%) and in Catalonia (−85.85%) [69]. One of the main reasons is the confinement imposed due to the high number of confirmed COVID-19 cases. It is worth mentioning that the differences between regions lie in three different factors, which will be analyzed throughout the investigation: seasonality (which will particularly affect the Balearic Islands), the origin of tourists, and the accessibility of each region (regarding the mobility restrictions due to the pandemic outbreak).

According to the INE [71], analyzing the different types of accommodation chosen by international tourists this year in comparison with 2019, hotels shows the strongest impact on the six leading touristic regions: Catalonia, Balearic Islands, Andalusia, Canary Islands, Community of Madrid, and Valencian Community, in that order. Regardless of the small difference between number of cases, the negative image of the country due to the high infection figures, had a high negative impact on international tourism.

**Figure 5:**
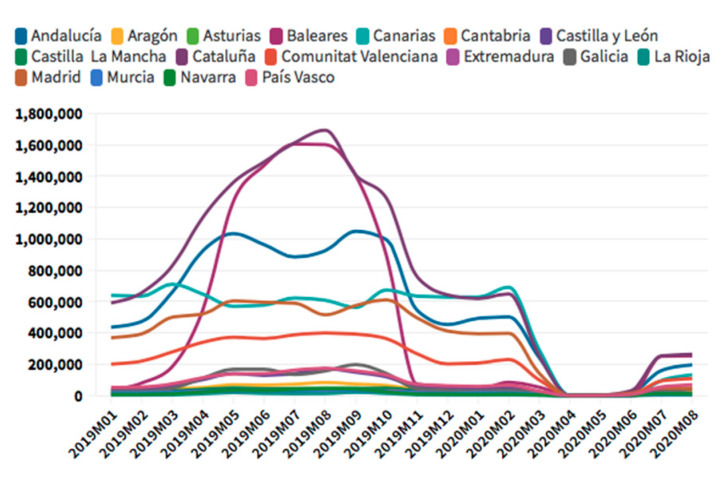
International tourism hotel occupancy. Source: Own elaboration from INE data.

**Figure 6:**
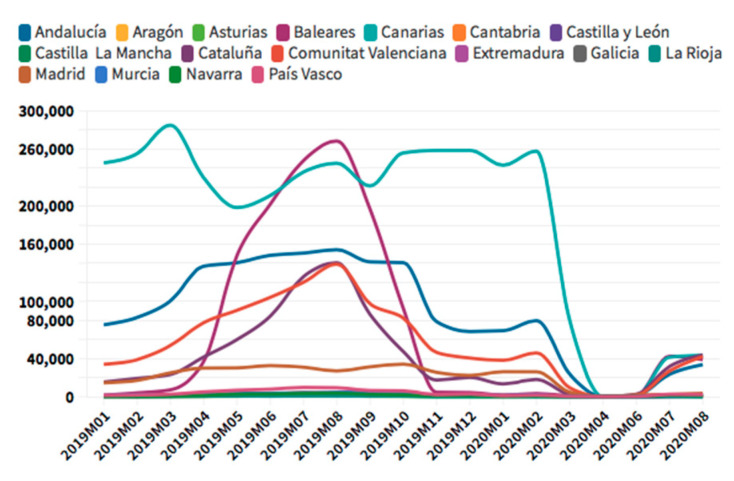
International tourism apartments occupancy. Source: Own elaboration from INE data.

**Figure 7:**
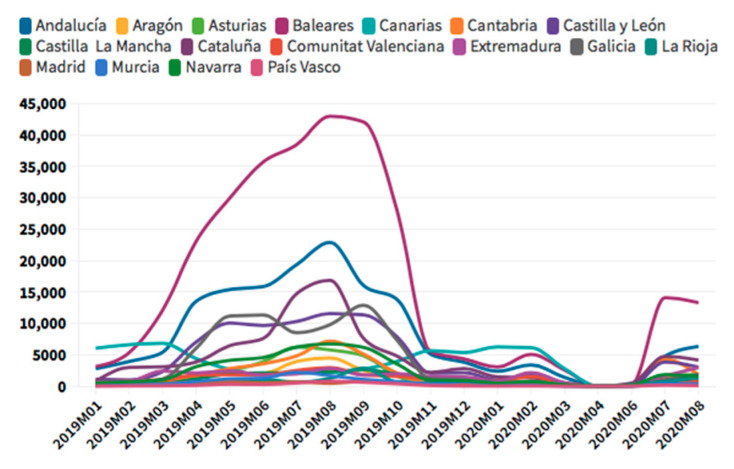
International tourism rural accommodation occupancy. Source: Own elaboration from INE data.

**Figure 8:**
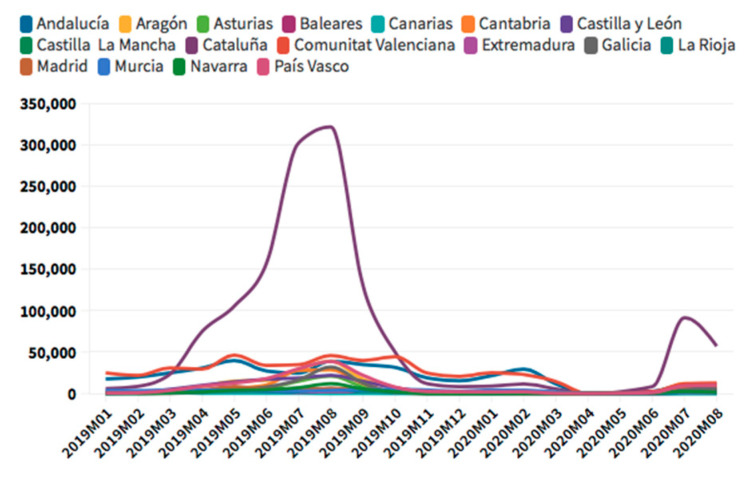
International tourism camping occupancy. Source: Own elaboration from INE data.

According to the Figure 5, Figure 6, Figure 7 and Figure 8, in the case of international visitors who stayed in tourist apartments, the evolution is quite similar to hotels. The greatest impact has been seen in the Community of Madrid, the Balearic Islands, and Andalusia. The impact of COVID cases in these regions must be considered in the first place, and second, the decrease in the accommodation supply of tourist apartments in many of the regions due to the restrictions nationwide. In the Balearic Islands, the evolution is very marked by seasonality, so the fall is already determined at the end of September 2019. On the other hand, other regions, such as the Canary Islands, the drop is noticeable already in February 2020. It should be considered that this region possesses a microclimate that allows stable visitors throughout the year.

The Balearic Islands was the leading region for international tourists in rural accommodation in 2019, which has decreased a 69% in August 2020, but still is the first region for this type of accommodation. Andalusia comes second, and Catalonia, the Valencian Community, the Canary Islands, and the Community of Madrid follow. This type of tourism has experienced relatively lower rates of decline; therefore, it represents an opportunity for the tourism in this critical situation.

As for international tourists who stayed in campsites, there is a wide difference between regions. There is a leading region par excellence in this type of accommodation, Catalonia. After Catalonia, Valencia and Andalusia are the regions with higher figures.

**Figure 9:**
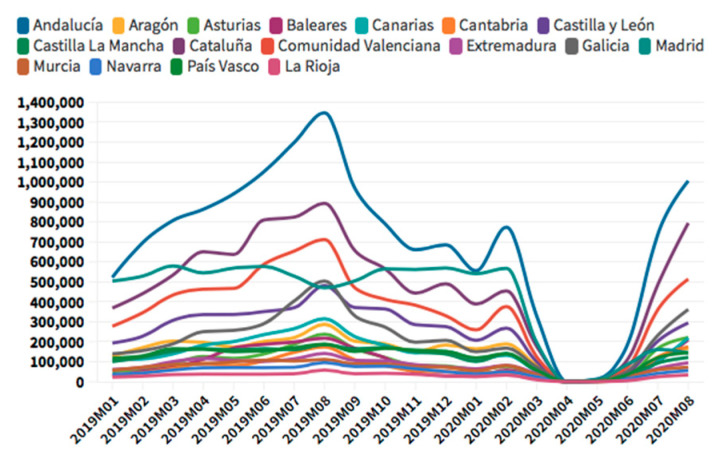
Domestic tourism hotels occupancy. Source: Self-made from INE data.

**Figure 10:**
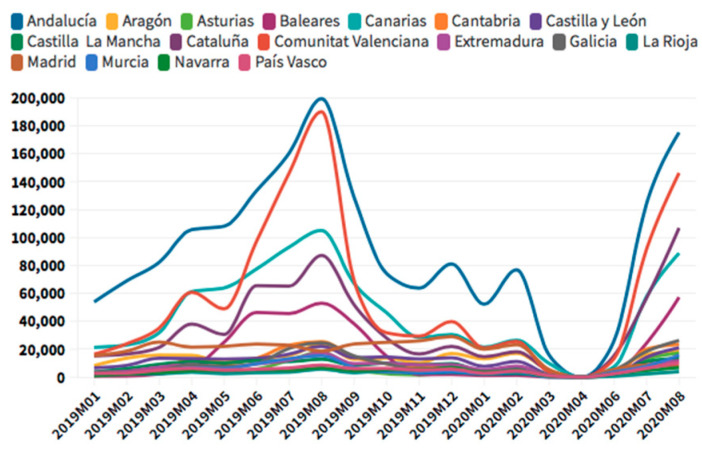
Domestic tourism apartments occupancy. Source: Self-made from INE data.

**Figure 11:**
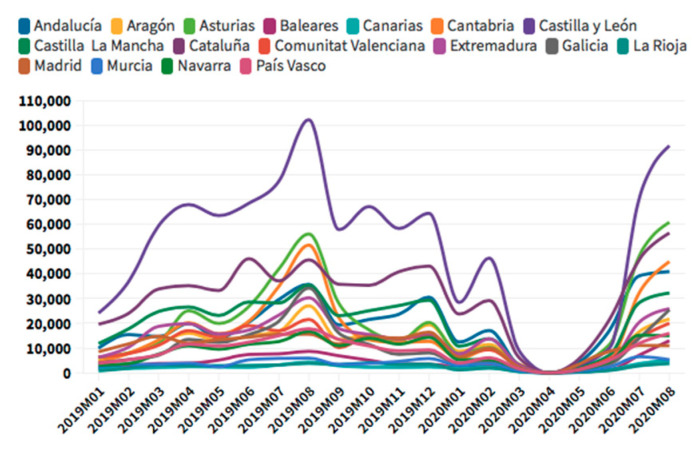
Domestic tourism rural occupancy. Source: Self-made from INE data.

**Figure 12:**
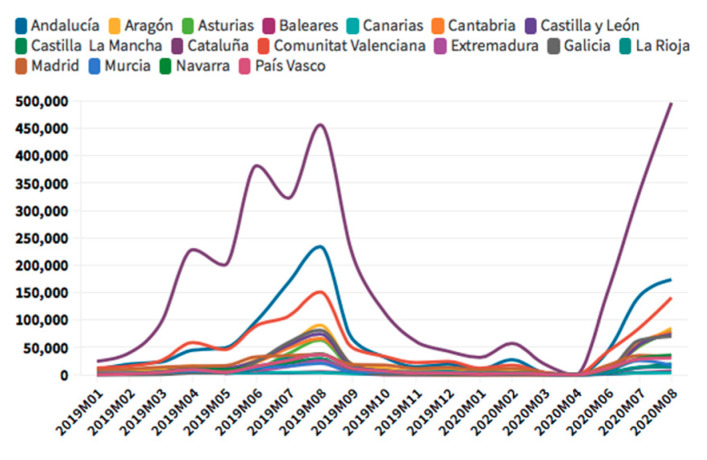
Domestic tourism camping occupancy. Source: Self-made from INE data.

According to Figure 9, Figure 10, Figure 11 and Figure 12, regarding domestic tourists who stayed in hotels, Andalusia was the leading region, followed by Catalonia and the Valencian Community. Other regions chosen were Galicia, Castilla and León, and the Community of Madrid, also showing high figures in domestic tourism, with a drop in number in August 2020, which are not as high as those experienced by international tourism.

The evolution of national tourists who stay in tourist apartments according to the INE [71] is slightly different from the hotel occupancy figures, nevertheless the fact that they have experienced a more fortuitous recovery it should be considered. The region with the highest figures for domestic tourism in touristic apartments in August 2019 was Andalusia. After Andalusia, the Valencian Community, the Canary Islands, and Catalonia, which would have experienced an increase in August 2020 in this type of accommodation compared to August 2019. This would be the first increase found in the series analyzed, so it shows how domestic tourism not only represents an opportunity for all regions, but the types of accommodation also play a key role. The Balearic Islands have also experienced a slight growth in tourist apartments, and Madrid have experienced a decline. In this last case, the fact that Madrid was the leading region in number of COVID-19 cases might have influenced in the tourists’ decisions.

The rural accommodation presents an even more encouraging evolution than international. Although in this case, the Balearic Islands is not the leading region, Castilla y León would be the first one instead. It should be noted that, although 2020 figures are lower than 2019, it shows a faster recovery than any other accommodation. As mentioned before, this type of tourism represents a great opportunity for the country, promoting a more sustainable tourism and helping tourism diversification for other regions that are not tourist leaders par excellence. In rural accommodation Catalonia manages to experience an increase in August 2020. Finally, it should be noted that the rest of the regions, traditionally leaders at the tourist level, such as: Andalusia, Valencian Community, Canary Islands, Balearic Islands, and Madrid, do not lead in this tourist accommodation. 

The evolution of domestic tourism in campsites, represents, together with rural accommodation, another key opportunity for the recovery of the Spanish tourism sector. In this tourist accommodation, Catalonia continues being the leader at international and domestic tourism. The figures show the highest increase of all accommodation types, which should serve as an example to the other regions in the implementation of tourist strategies. After Catalonia, at a certain distance, are Andalusia and the Valencian Community. Despite the lower figures in comparison with the first three regions, Asturias and the Balearic Islands have experienced a growth from August 2019 to August 2020 in this type of accommodation for domestic tourism.

## 6. Discussion

Throughout this investigation, we have seen how from January to July 2020 Spain registered 321,561 COVID-19 cases [64]. The regional distribution of these infections means that the highest rate per 100,000 inhabitants was in Andalusia, Catalonia, Valencia, Madrid, the Canary Islands, and the Balearic Islands; these regions are also those with the highest tourist flows in Spain [63] and are the most dependent on the tourist sector. This finding therefore confirms Proposition 1, which states that the regions with more confirmed cases are those most affected by the pandemic.

Proposition 2 points out that the most touristic regions of Spain will be the most affected by the crisis. We found that regions with high rates of COVID cases were Andalusia, Catalonia, Valencia, the Canary Islands, and the Balearic Islands. These regions are also the most dependent on tourism for their economy. For example, they have cited the case of the Canary Islands which presented, in the period analyzed (from January 31 until November 2020) a 61.4% decrease in GDP compared to the same period in 2019. Catalonia had a decrease of 75.6%; Andalusia a decrease of 72.3%; in the Valencian community the decrease was 69.7%; and 67.1% in the Community of Madrid. Of all of them, the Balearic Islands was the region most affected by the crisis in terms of the decrease in tourist visitors with −87% [69].

On the other hand, we can see that the regions most affected by this pandemic, in the first and second waves, have been the most dependent on international tourism, but also on their seasonal flows (related to summer and good weather), such as Catalonia, Valencia, the Canary Islands, and the Balearic Islands. These regions register the lowest GDP figures, showing a clear relationship between the high rates of cases and the decrease in visitors to these regions, which confirms Proposition 3, which stated that the most infected regions would be the least visited; related at the same time with Proposition 1.

In relation to Proposition 4, as shown in Figure 2, the most touristic regions have higher unemployment rates. Available data shows that the highest unemployment figures, in the second quarter of 2020, are found in the six most touristic regions representing 69.25% of total Spanish unemployment. At the same time, these regions register 70.03% of total unemployment in the service sector nationwide. In line with the previous finding, it has been verified that the regions with more cases are those with the highest unemployment rates (Proposition 5).

Finally, this work shows the importance of domestic tourism alleviating the ravages of the health and socioeconomic crisis, with rural and camping types of accommodation leading the visitors’ choices. This evidence one of the most appropriate options for the development of sustainable tourism, providing quality of life for residents and safety for tourists (Proposition 6). We have seen how the data shows a faster recovery of domestic tourism than the experienced in international tourism, which in our opinion is an opportunity for regions that are not tourism leaders, although they have been developing alternative offers linked to rural tourism; these regions are for example Castilla y León, Asturias, and Cantabria, which in this pandemic have focused their strategies on the promotion of domestic tourism (Proposition 6). In turn, the Spanish regions that traditionally lead the tourism sector, such as Andalusia, Valencia, the Canary Islands, the Balearic Islands, and Madrid, have not developed many alternative offers to mass tourism. In this context, experiential, rural and active tourism represents one of the strong points of the sector; the accommodations associated with these tourism practices are the ones that have recovered the fastest and have experienced relatively lower rates of decline. This type of tourism offers opportunities for the regions most affected by the impacts of the pandemic, helping to diversify the tourism activity, especially in those areas suffering from high seasonality levels.

Some implications can be drawn from the data analyzed in this article. The current moment, in the collapse of the tourism model in Spain, which we trust is conjunctural can help to develop certain implications, is the time to continue moving towards a more sustainable tourism model from an economic, social, and environmental point of view. The post-pandemic period could align the tourism industry more closely with the Sustainable Development Goals (SDGs) and lead to a greener, more inclusive and resilient industry that provides decent work for all tourism workers. It also has implications for safety and health, including for the national health system, which is key for all destinations. This should be reflected in their marketing and communication strategies, by planning procedures to regain the confidence of potential visitors, developing protocols to ensure health safety, to ensure the competitiveness of the tourism industry and guarantee the safety of all. There are also implications in terms of communication: Government communication strategies must be aligned with these solutions in order to obtain maximum benefits. As an example, the domestic tourism campaign that Spain has launched for the first time in history, ‘#DescubreLoIncreíble’ [72], which is being promoted on social networks. This type of communication campaign should target rural accommodations and campsites to help the tourism sector.

## 7. Conclusions

In a pandemic context, tourists are increasingly opting for nearby environments, if not their own countries, rather than for grand tours to distant and relatively unsafe places. This change in tourists’ perspective could lead them to avoid overcrowded and mass tourism destinations in favor of more familiar, but less populated and more reliable places. The evolution of domestic tourism in Spain could be a great hope for the country resilience and, with the right measures, it could favor a change in the tourism development model of countries like Spain, focusing more on sustainable, less intensive proposals with less impact on the population and the environment. This new “era of tourism” involves in a more decisive way regions where until now mass “sun and beach” tourism has been dominant.

This study shows that the impact of the pandemic is still being evaluated. The analysis of the relationship between the tourism sector and the pandemic outbreak in Spain provides an instructive case study to assist the sector in the recovery process.

Our objective was to study the effects of the spread of the virus in the different regions and its impact on the economy and regional tourism flows. To this end, we have been guided by a set of propositions that we have tried to demonstrate with the results of the study. In essence, we have seen how the impact of the pandemic has been greater in the regions with higher rate of contagion, and this, in the period studied, coincides with the regions that are most dependent on tourist flows. Those regions that have invested in a rural and alternative tourism development supporting, for example, rural and camping lodgings, have experienced a smaller decrease. This evidence should be considered in tourism plans and strategies. This type of tourism generates high profits and allows preserving the physical distance required in this health crisis.

## Figures and Tables

**Table 1:** Regions most impacted by the Covid-19 crisis.

	Unemployment	Unemployment Service Sector	GDP	GDP	COVID-19
			Q1 2020	Q2 2020	Cases
*Andalusia*	980.096	648.584	−5.25%	−15,6%	19.279
*Catalonia*	485.019	360.556	−5.19%	−22%	77.43
*Valencian Community*	456.796	309.153	−5.11%	−22.1%	17.298
*Community Madrid*	417.199	334.354	−5.33%	−18%	78.056
*Canary Islands*	261.714	204.2	−5.45%	−21%	2.726
*Balearic Islands*	74.293	58.581	−5.55%	−26.4%	2.849
*TOTAL*	2675.117	1915.428	−5.24%	−18,5%	197.638

Source: Own elaboration from SEPE, INE, and CNE data.

## Data Availability

The data that support the findings of this study are available from the corresponding author, upon reasonable request.
